# 

**Published:** 1899-05

**Authors:** 


***NOTICE.—This JOURNAL and THE INTERNATIONAL JOURNAL OF SURGERY one year for $1.00 cash, to new subscribers. Out-of-town checks not accepted unless 10c. is added for cost of collection.***

THE Secretary of the State Medical Examining Board, Dr. E. R. Anthony, informs us that the next meeting of this Board for the purpose of examining candidates will be held in the Capitol building, in Atlanta, on May 10th, 1899.

---

***NOTICE.—This JOURNAL and THE INTERNATIONAL JOURNAL OF SURGERY one year for $1.00 cash, to new subscribers. No out-of-town checks accepted unless 10c. is added for cost of cashing.***

### *REPRINTS RECEIVED.*

---

Appendicitis. By H. O. Walker, M.D., Detroit, Mich.

Suggestions and Criticisms. By Orpheus Everts, M.D., Cincinnati.

Twenty-fifth Annual Report of the Superintendent of the Cincinnati Sanitarium for year 1898.

Diphtheria. By W. D. Travis, A.B., M.D., Covington, Ga.

Growing Children: Their Clothes and Deformity. By E. Noble Smith, F.R.C.S., London.

The Progress of Rhino-Laryngology. By W. Scheppegrell, A.M., M.D., New Orleans.

1. Adeno-Carcimona of the Nose, with Report of a Case. 2. A Nasopharyngeal Polypus of Enormous Size. By Max Thorner, M.D., Cincinnati.

Headaches, Causes and Treatment, with Especial Reference to Nasal and Ocular Headaches. By A. D. McConachie, M.D., Baltimore.

A Review of Dr. William Beaumont's Experiments on Alexis St. Mortier. By S. C. Ayers, M.D., Cincinnati.

Anterior Poliomyelitis or Infantile Paralysis. By Chas. A. Lahenberg, M.D., Richmond, Va.

Hernia. Clinical Lecture delivered at the New York Post-Graduate Medical School. By W. B. De Garmo, M.D., New York.

The Etiology of Phthisis. By W. Gardner, M.D., New York.

The Bête Noire of the Vocalist. By Edwin Pynchon, M.D., Chicago.

The Absolute and Permanent Cure of Tonsillitis. By Edwin Pynchon, M.D., Chicago.

Annual Reports of the Bureau of Health of the City of Denver for the years 1897–98.

The Shape and Position of the Stomach. By Henry Wald Beltman, B.S., M.D., Cincinnati.

